# Why does a mitochondrion need its individual cristae to be functionally autonomous?

**DOI:** 10.1080/23723556.2019.1705119

**Published:** 2020-01-10

**Authors:** Marc Liesa

**Affiliations:** a Department of Medicine, Endocrinology, David Geffen School of Medicine at UCLA, Los Angeles, CA, USA; b Molecular Biology Institute at UCLA, Loa Angeles, CA, USA

**Keywords:** Mitochondria, cristae, membrane potential, cristae junction

## Abstract

We demonstrated that individual cristae in one mitochondrion function as autonomous electrochemical units, with this autonomy being maintained by cristae junction modulators. Here, I will summarize our novel findings, discuss the advantages of cristae insulation and reconcile this newly characterized cristae autonomy with previous studies showing coordinated cristae function.

The difference in electric potential generated by the proton gradient across the mitochondrial inner membrane (ΔΨm) is used to transform the energy released from nutrient catabolism to ATP. However, ΔΨm is also required for influx/efflux of metabolites feeding anabolic reactions and activating stress-responses, as well as for the import of nuclear-encoded mitochondrial proteins. The dependency on ΔΨm shared by these processes and the organization of the inner membrane (IM) in cristae raise these questions:

- How does a single mitochondrion organize the use of the energy contained in the ΔΨm to fuel and/or prioritize different competing processes?
- Why the abrogation of cristae junction (CJ) modulators decreases ΔΨm and impairs electron transport chain (ETC) function?

ΔΨm was demonstrated to be continuous along the IM, as photodamaging a fraction of one mitochondrion causes concurrent ΔΨm dissipation in proximal and distal areas from the site of photodamage.^1^ Consequently, it seemed unlikely that cristae formation allowed compartmentalizing the IM to regions with distinct ΔΨm. In contrast, topological studies of the ETC components and ATP synthase challenged the absence of ΔΨm heterogeneity along the IM. ETC complexes are located in the lateral surfaces of cristae and ATP synthase is located at cristae edges.^2,3^ Furthermore, the inner boundary membrane (IBM) is the preferred location of protein import^2,4^ (Figure 1). With this heterogeneous distribution of ΔΨm-generating and consuming processes, it was possible that ΔΨm could be different in distinct areas of the IM.

**Figure 1.**
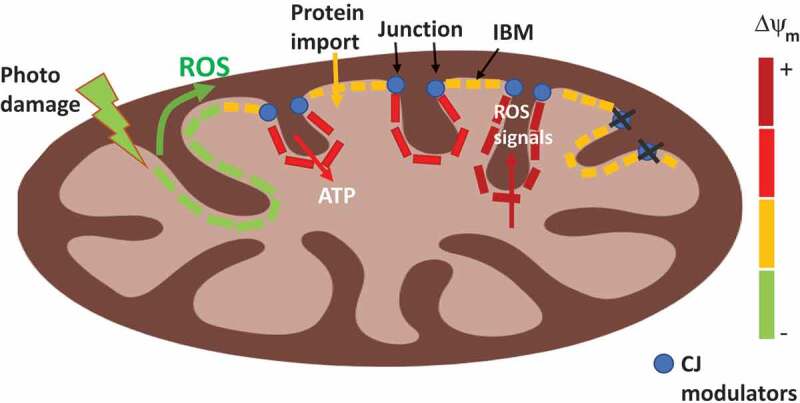
**Individual cristae in a mitochondrion are functionally autonomous**. The difference in membrane potential generated by the proton gradient (ΔΨm) in each crista is color-coded with red to green ratio: red-hyperpolarized, yellow-polarized, green-depolarized. Cristae junction (CJ) modulators are depicted as blue dots. Individual cristae and the inner boundary membrane (IBM) in one mitochondrion can have different ΔΨm, as ΔΨm might be serving distinct mitochondrial processes: ATP synthesis, reactive oxygen species (ROS)-mediated signaling or protein import. Deleting CJ modulators reduce the difference in ΔΨm between crista and the IBM. The wave-like pattern of depolarization-induced by photodamage could be explained by a diffusible signal (ROS) and/or self-propagating signal (lipid peroxides) that causes depolarization in distal sites. The need for such a diffusible/self-propagating signal reflects the need to sense unrepairable damage and send irreversibly injured mitochondria to mitophagy.

By using super-resolution microscopy and four mitochondrial dyes, Nonyl Acridine Orange (NAO), Tetramethylrhodamine ethyl ester (TMRE), Mitotracker-green (MTG) and Rhodamine-123, we visualized individual cristae in respiring cells.^5^ These dyes require ΔΨm to accumulate in mitochondria. However, NAO and MTG do not properly report on ΔΨm, as when they reach mitochondria, they stay bound in a ΔΨm-independent manner.^6,7^ Only Rhodamine-123 and TMRE report on ΔΨm real-time.^6,7^ We confirmed that TMRE reported ΔΨm in cristae, as cristae TMRE was dissipated by photodamage, mitochondrial uncoupling, and spontaneous depolarization.^5^ TMRE dissipation occurred without changes of cristae MTG signal, further proving ΔΨm-sensitivity of cristae decoration by TMRE.^5^ Then, why diffusible TMRE preferentially labeled cristae? We concluded that this preference was reflecting a close juxtaposition of TMRE to the matrix-side of the IM, caused by the highest ΔΨm values expected in closer apposition to the IM.

A challenge using TMRE to compare ΔΨm in cristae versus the IBM is that cristae membrane composition is different. Therefore, a higher fraction of TMRE molecules could be decorating membranes in a non-Nernstian manner (binding) in cristae, when compared to the IBM. To address this, we measured the response of the difference in TMRE fluorescence between the IBM and cristae to compounds acutely changing ΔΨm, such as the ATP synthase inhibitor oligomycin.^5^ If elevated TMRE signal in cristae was exclusively caused by increased binding to membranes, acute treatment with oligomycin should not change the difference in TMRE signal between cristae and IBM. Supporting that ΔΨm was elevated in cristae (Figure 1), oligomycin exacerbated the difference in TMRE labeling between cristae and IBM.^5^ Then, we asked whether CJ modulators could be responsible for maintaining higher ΔΨm in cristae, as deleting some CJ modulators disrupt cristae structure, oxidative phosphorylation activity and widen CJs.^8,9^ We found that knocking-out either mitochondrial dynamin like GTPase (Opa1) or different components of the Mitochondrial Contact Site and Cristae Organizing System (MICOS), was associated with a decrease in the difference of ΔΨm between cristae and IBM. Furthermore, the abrogation of mitochondrial dynamics-dependent quality control, through co-deletion of Opa1 & Dynamin 1 like (Dnm1l, aka Drp1), induced the formation of cristae vesicles detached from the IBM.^5^ These results demonstrated that cristae can become even physically independent from the IBM. We speculate that the formation of cristae vesicles could be revealing an uncharacterized recycling process of mitochondrial components controlled by CJ modulators. A defect in this recycling process, and thus in mitochondrial quality control, could help explaining decreased ETC activity induced by the abrogation of CJ modulators and cristae structures.

How cristae autonomy is reconciled with ΔΨm continuity along the IM^1^? Studies demonstrating concurrent ΔΨm depolarization in proximal and distal areas from the photodamage site had a low temporal resolution, obtaining the first ΔΨm measurements 5 s after photodamage.^1^ Thus, it was possible that signaling events leading to depolarization could be completed in less than 5 s. To address this, we measured ΔΨm every 0.15 s after photodamage and found that ΔΨm depolarization showed a wave-like pattern, starting at the site of photodamage and spreading to distal areas.^5^ This wave-pattern supports that photodamage generates a diffusible and/or self-propagating signal that induces distal depolarization. We speculate that this signal could be reactive oxygen species (ROS) and/or self-propagating lipid peroxides (Figure 1). The existence of such a signal would allow sensing unsolvable damage and communicate the need to eliminate the injured mitochondrion, as depolarization tags mitochondria for removal by mitophagy.^10^ If such damage occurred in a long mitochondrion, fragmentation into smaller mitochondria would allow to segregate damaged areas and selectively tag them for mitophagy. Undamaged areas would form units that could recover ΔΨm and escape from mitophagy. Consequently, the connection of ΔΨm cristae by diffusible signals inducing depolarization is explained by i) the need to remove an entire mitochondrion by mitophagy to ensure quality control and ii) depolarization being the signal selected to eliminate mitochondria by mitophagy.

Cristae autonomy could also allow compartmentalizing the energy within ΔΨm in the IM, to avoid competition for ΔΨm consumption of concurrent mitochondrial processes. IBM insulation permits dedicating ΔΨm in IBM mostly to support protein import. Then, the electrical insulation of one crista from the IBM and from other cristae would let one crista to retain the hyperpolarization needed to produce ROS for signaling, while other cristae could concurrently consume ΔΨm to fuel ATP production (Figure 1).

We believe our work has provided important evidence explaining why cristae morphology and junctions are key determinants of mitochondrial function in health and disease.

## References

[1] Amchenkova AA, Bakeeva LE, Chentsov YS, Skulachev VP, Zorov DB. Coupling membranes as energy-transmitting cables. I. Filamentous mitochondria in fibroblasts and mitochondrial clusters in cardiomyocytes. J Cell Biol. 1988;107(2):1–3. doi:10.1083/jcb.107.2.481. 3417757 PMC2115217

[2] Vogel F, Bornhovd C, Neupert W, Reichert AS. Dynamic subcompartmentalization of the mitochondrial inner membrane. J Cell Biol. 2006;175(2):237–247. doi:10.1083/jcb.200605138. 17043137 PMC2064565

[3] Strauss M, Hofhaus G, Schroder RR, Kuhlbrandt W. Dimer ribbons of ATP synthase shape the inner mitochondrial membrane. Embo J. 2008;27(7):1154–1160. doi:10.1038/emboj.2008.35. 18323778 PMC2323265

[4] Horvath SE, Rampelt H, Oeljeklaus S, Warscheid B, van der Laan M, Pfanner N. Role of membrane contact sites in protein import into mitochondria. Protein Sci. 2015;24(3):277–297. doi:10.1002/pro.2625. 25514890 PMC4353355

[5] Wolf DM, Segawa M, Kondadi AK, Anand R, Bailey ST, Reichert AS, Bliek AM, Shackelford DB, Liesa M, Shirihai OS Individual cristae within the same mitochondrion display different membrane potentials and are functionally independent. Embo J. 2019;38(22):e101056. doi:10.15252/embj.2018101056. 31609012 PMC6856616

[6] Presley AD, Fuller KM, Arriaga EA. MitoTracker Green labeling of mitochondrial proteins and their subsequent analysis by capillary electrophoresis with laser-induced fluorescence detection. J Chromatogr B Analyt Technol Biomed Life Sci. 2003;793(1):141–150. doi:10.1016/S1570-0232(03)00371-4. 12880861

[7] Farkas DL, Wei MD, Febbroriello P, Carson JH, Loew LM. Simultaneous imaging of cell and mitochondrial membrane potentials. Biophys J. 1989;56(6):1053–1069. doi:10.1016/S0006-3495(89)82754-7. 2611324 PMC1280610

[8] Rabl R, Soubannier V, Scholz R, Vogel F, Mendl N, Vasiljev-Neumeyer A, Körner C, Jagasia R, Keil T, Baumeister W, et al. Formation of cristae and crista junctions in mitochondria depends on antagonism between Fcj1 and Su e/g. J Cell Biol. 2009;185(6):1047–1063. doi:10.1083/jcb.200811099. 19528297 PMC2711607

[9] Cogliati S, Frezza C, Soriano ME, Varanita T, Quintana-Cabrera R, Corrado M, Cipolat S, Costa V, Casarin A, Gomes L, et al. Mitochondrial cristae shape determines respiratory chain supercomplexes assembly and respiratory efficiency. Cell. 2013;155(1):160–171. doi:10.1016/j.cell.2013.08.032. 24055366 PMC3790458

[10] Twig G, Elorza A, Molina AJ, Mohamed H, Wikstrom JD, Walzer G, Stiles L, Haigh SE, Katz S, Las G, et al. Fission and selective fusion govern mitochondrial segregation and elimination by autophagy. Embo J. 2008;27(2):433–446. doi:10.1038/sj.emboj.7601963. 18200046 PMC2234339

